# A Demonstration of Long-Term Outcomes in a Case of Toe to Thumb Transfer Following Traumatic Amputation

**DOI:** 10.7759/cureus.12576

**Published:** 2021-01-08

**Authors:** Jake Melhuish, Laetitia Jervis, Kess X Melhuish

**Affiliations:** 1 General Physician, Royal Army Medical Corps, Oxford, GBR; 2 Paediatrics, University of East Anglia, Norwich, GBR; 3 Plastic and Reconstructive Surgery, University Hospitals of Leicester National Health Service Trust, Leicester, GBR

**Keywords:** thumb, transplant, toe, outcomes

## Abstract

The thumb is pivotal to many functions of the hand. Loss or absence of the thumb can be catastrophic to a patient’s functioning. Different methods can be employed to surgically fashion a new thumb. This case report aims to demonstrate one patient’s 34-year experience, reporting objective measures for a toe to thumb transfer, and documenting aesthetic outcomes. A semi-structured interview was conducted to gain insight into the patient's personal experiences. Validated assessment tools were used to assess range of movement and power, including Kapandji test, Disabilities of the Arm, Shoulder and Hand (DASH) questionnaire, Cochin Hand Function Scale, Qingfeng Hand Dynamometer, Self-Administered Foot Evaluation Questionnaire (SAFE-Q). A physical examination on the neothumb was performed, along with photography to document aesthetic outcome. A 55-year-old white British male injured his right thumb whilst on a placement year in a sugar refinery in the Netherlands. The thumb was traumatically amputated proximal to the metacarpophalangeal joint. The patient lived for a year without a thumb on his dominant hand, which had a huge effect on his functioning, with extensive input from physiotherapists to help increase his handgrip strength. He then underwent a transfer operation, with the removal of the second toe which was transferred to create a right neothumb. The patient has high physical functioning of his neothumb, however, he is still mildly limited due to pain 34 years post-operation. The objective assessment tools demonstrate a high functioning of the neothumb, with only mild deficits in the dexterity and the physical functioning of the right hand. He experiences mild pain and reduced function of the donor foot. Light touch was found to be absent on the right thumb, but present on the left. Two-point discrimination was 7 mm on the right thumb, and 0.5 mm on the left. In this case, we present a toe to thumb transfer that had very good outcomes after 34 years with few complications. This case demonstrates that great adaptation can occur over long periods of time, restoring near-normal function. Measures of functional outcomes were generally high, with main deficiencies in fine motor skills such as picking up a penny from a flat surface and undoing small buttons. Additionally, there can be good long term outcomes from toe to thumb transfer despite moderate impairment of function. There is hope that this can be used to encourage and reassure patients and surgeons alike that the neothumb is likely to give good outcomes both functionally and aesthetically for many years.

## Introduction

The thumb is pivotal to many functions of the hand; these include pinching, gripping, and enclosing the palm. Hence, loss or absence of the thumb, from distal to metacarpophalangeal joint, of either hand has the possibility to be catastrophic to a patient’s functioning. If replantation is not possible then surgically fashioning a new thumb must be explored. There are four ways described in the literature in which this can be achieved: transfer of the second toe or the great toe, a great toe wrap-around, or a trimmed great toe [1]. All these variants have been shown to have excellent success rates of between 95% and 100% [1]. The best outcomes for appearance and function are generally believed to be with the great toe transfer [2]. The wrap-around and trimmed variations of the great toe transfer were developed to reduce donor morbidity and increase aesthetic outcomes. Although attempts have been made to achieve consensus for the type of surgery performed, transfer technique is mostly based on a surgeon’s preference and training.

There is a paucity of evidence for outcomes of patients with toe to thumb transfers beyond five years. This case report aims to demonstrate one patient’s 34-year experience, reporting objective measures for a second toe to thumb transfer, and exploring aesthetic outcome.

## Case presentation

Methods

Informed verbal consent was obtained from the patient.

Interview

A semi-structured interview was conducted in August 2020, to gain insight into the patient's personal experiences over the last 34 years.

Assessment Tools

**Figure 1:**
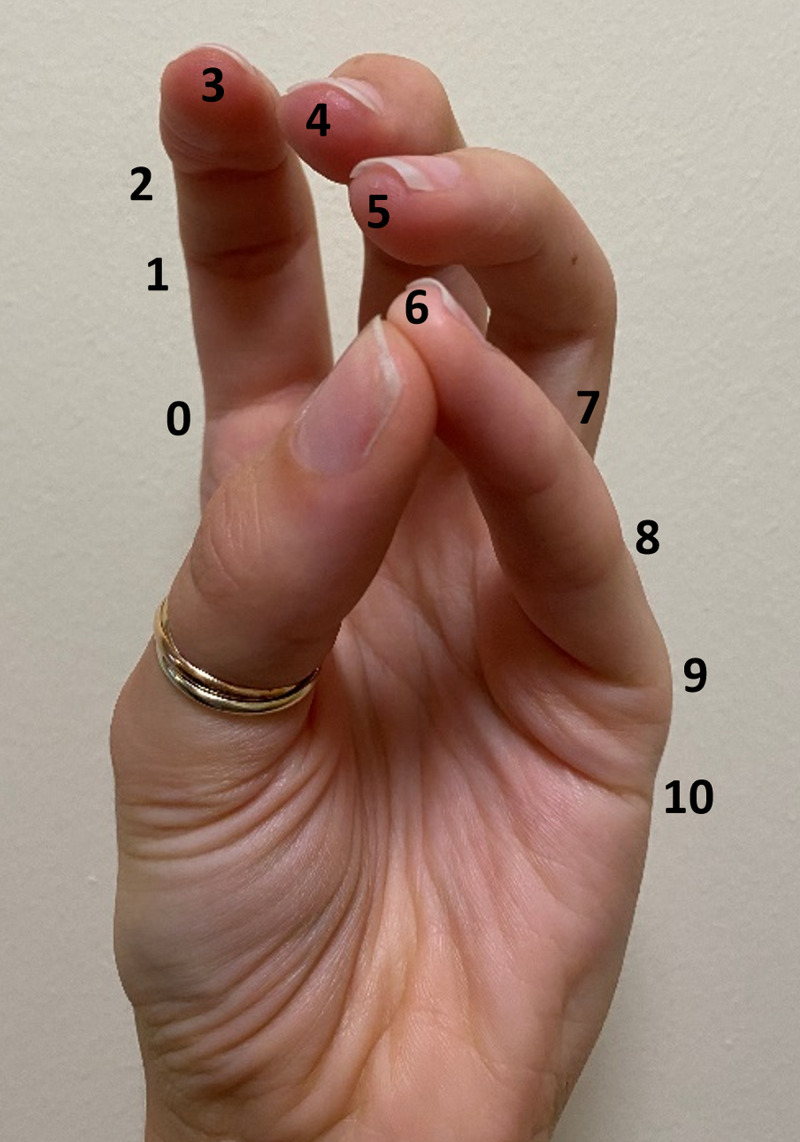
Kanpandji score, a test of thumb opposition. A score of 0 demonstrates no opposition, a score of 10 demonstrates maximal opposition.

Validated assessment tools, using indicators for range of movement and power, were performed:

1. The Kapandji test is a measure of motor-ability and dexterity in the hand, using thumb opposition. The patient is required to touch the tip of their thumb to points on the same hand to generate a score out of 10 [3], as can be seen in Figure 1.

2. The Disabilities of the Arm, Shoulder and Hand (DASH) questionnaire is an upper-limb specific outcome measure, evaluating a patient’s self-reported disability and symptoms, based on the performance of physical tasks. There are 30 questions, giving a score out of 100, with zero being no disability and 100 being the most severe disability [4].

3. The Cochin Hand Function Scale is a questionnaire used to assess the functional ability of the hand, using 18 questions about daily activities. Each question is scored from zero to five, generating a score out of 90, with zero being no disability and 90 being the most severe disability [5].

4. The Qingfeng Hand Dynamometer was used to test the hand grip strength (HGS) in both the affected and unaffected hand, measured in kilograms. The patient was seated, with their elbow by their side and flexion at the elbow, with the wrist in a neutral position [6].

5. The Self-Administered Foot Evaluation Questionnaire (SAFE-Q) is an ankle and foot-specific outcome measure, assessing patient’s symptoms, physical function, and quality of life with pathological conditions of the ankle and foot. The questionnaire consists of 34 questions in five subsets, each subset providing a separate score out of 100. There are nine optional questions regarding sports activity, providing another score out of 100 [7].

A physical examination was performed on the thumb of both the right and the left hand. Both light touch and pain were tested, as well as two-point discrimination. Photography of the affected hand and foot was used to document the aesthetic outcome.

Results

Interview

The patient is a right hand dominant, 55-year-old white British male, currently employed in a desk-based job as a Sales Director. There is no history of manual labor employment. He attended University, achieving a higher-level education qualification. Currently, the patient is fully independent with their activities of daily living. He possesses both a category B1 driving license and a forklift truck driving license. He participates in outdoor swimming events, sprint triathlons, and racket sports.

The patient’s injury occurred on November 6th, 1984 whilst on an industrial placement in a sugar refinery in the Netherlands. He was removing sugar beet with his right hand, from underneath an Archimedes’ screw. The screw removed the right thumb proximal to the metacarpophalangeal joint. The injury was classified as traumatic amputation.

The patient spent one week in a hospital in the Netherlands. The amputated thumb was recovered, however was deemed to be too contaminated with organic waste to be reattached. Under local anesthetic, the bone stump on the right hand was clipped, and a skin flap stitched over the wound. The patient then returned to the United Kingdom and the wound took one month to heal.

The patient spent one year without a thumb on his right hand. On questioning, he stated that he would have rated the pain a 10 out of 10 almost constantly, with the pain being made worse by the cold, or if knocked against a hard surface. He returned to the University of Bradford, two months after the injury. He had to learn to write with his left hand, unsuccessfully, so resorted to writing with the pen in his right hand, between his index and middle fingers. This severely affected the patient’s studies as he was unable to take notes quickly enough in lectures, resulting in him not attending. There were a number of tasks the patient struggled to perform, including picking things up, putting a key in a door and turning it, riding a bike, doing up the zip on trousers, doing up buttons, and driving.

Throughout this year, the patient received input from a physiotherapist. The exercises concentrated on strengthening the remaining digits on the right hand, whilst also increasing overall hand-grip strength.

The injury impacted the patient psychologically, as he referred to it as a ‘deformity’. The patient felt extremely self-conscious of this and therefore tried to keep it hidden. He found gestures such as shaking hands embarrassing, as the recipient would expect pressure to be applied by the thumb. The patient consequently wore gloves to hide the injury.

Nine months following the injury, the patient was offered two operations to surgically fashion a new thumb: the great toe transfer, and transplant of the index finger to the thumb position. The patient declined both operations as he felt that the risks outweighed the benefits. He was then put into contact with Mr. Barry Jones, who was a trainee plastic surgeon at the time and offered the second toe to thumb transfer operation. The patient accepted this operation, which went ahead in November 1985. Prior to the operation, the patient had to give up smoking due to the negative effects on vascular supply and the healing process.

The patient remained in the hospital for 10 days following the operation. He recalled the recovery process as being ‘slow and painful’. The wound took many months to heal, with a lot of pain associated with this, particularly from the skin graft site on the thigh. Six weeks postoperatively, the patient was allowed to start mobilizing the thumb. Physiotherapists were involved in the mobilizing process, aiming to strengthen the muscles in the thumb and the hand, by using passive movements. After six months of physiotherapy, the patient made the first active movement of his new thumb. It took nine months for touch sensation to be present in the thumb, along with the perception of hot and cold. However, the obtained touch sensation only involves contact, the patient is unable to determine between two different textures.

The patient stated that even now, 34 years post-operation, he still has to concentrate on movements of the thumb. The site on the foot where the index toe was removed from is still very sensitive to the cold, resulting in adaptions in the patient’s home, such as heated flooring, being required.

Assessment Tools

Table 1 shows the results obtained from the validated assessment tools that were performed on the patient.

**Table 1:** Results table for assessment tools

Assessment Tool		
Kanpandji Test (out of 10)	Right Hand	Left Hand
	7	10
Disabilities of the Arm, Shoulder and Hand Questionnaire (out of 100)	19.2	
Cochin Score (out of 90)	38	
Qingfeng hand dynamometer	Right Hand	Left Hand
	90kg	75kg
Self-Administered Foot Evaluation Questionnaire (each item out of 100)	Pain and Pain-Related	78
	Physical Functioning and Daily Living	97.73
	Social Functioning	95.83
	Shoe-Related	83.33
	General Health and Well-Being	100
	Optional Item: Sports Activity	86.67

Physical Examination

It was found that light touch was absent when examined on the right thumb, however fully intact on the left thumb. Two-point discrimination was found to be 7 mm on the right thumb and 0.5 mm on the left.

Photographs

**Figure 2:**
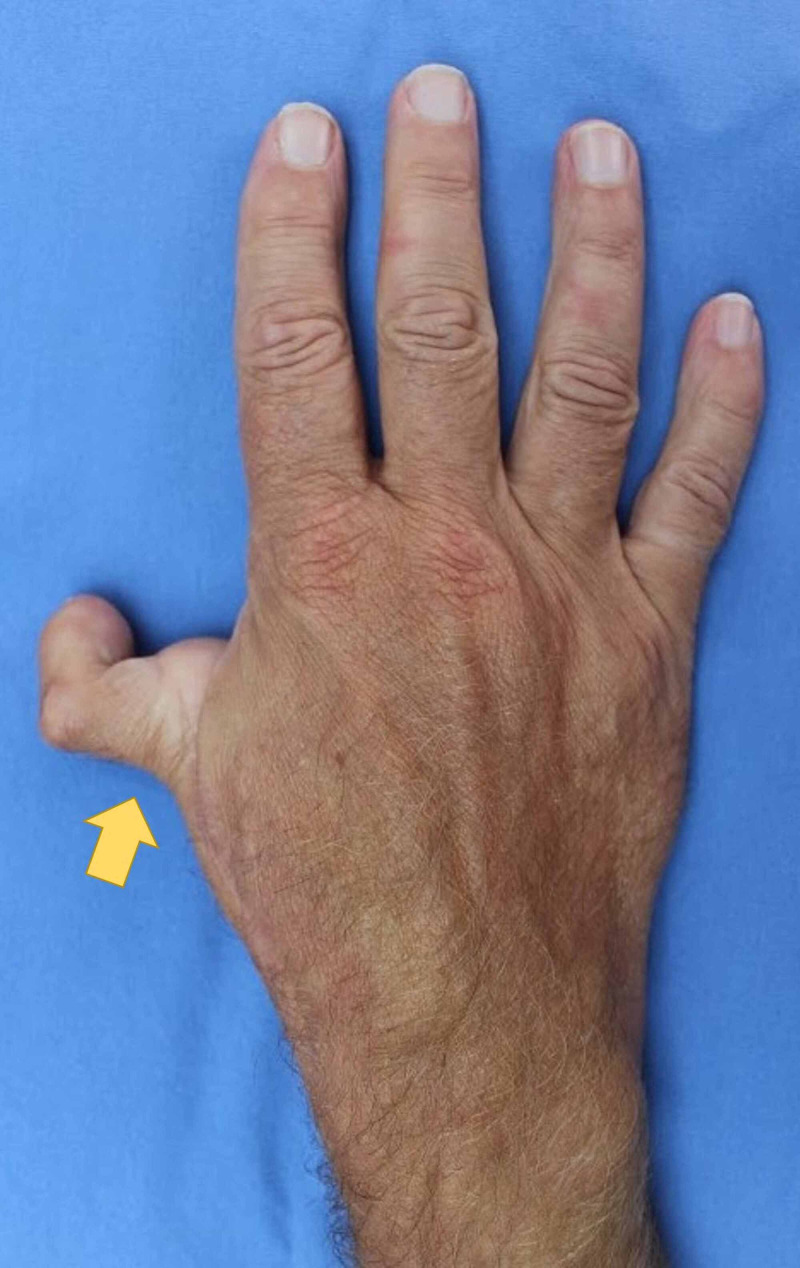
The dorsal surface of the right hand, demonstrating the dorsal aspect of the neothumb

**Figure 3:**
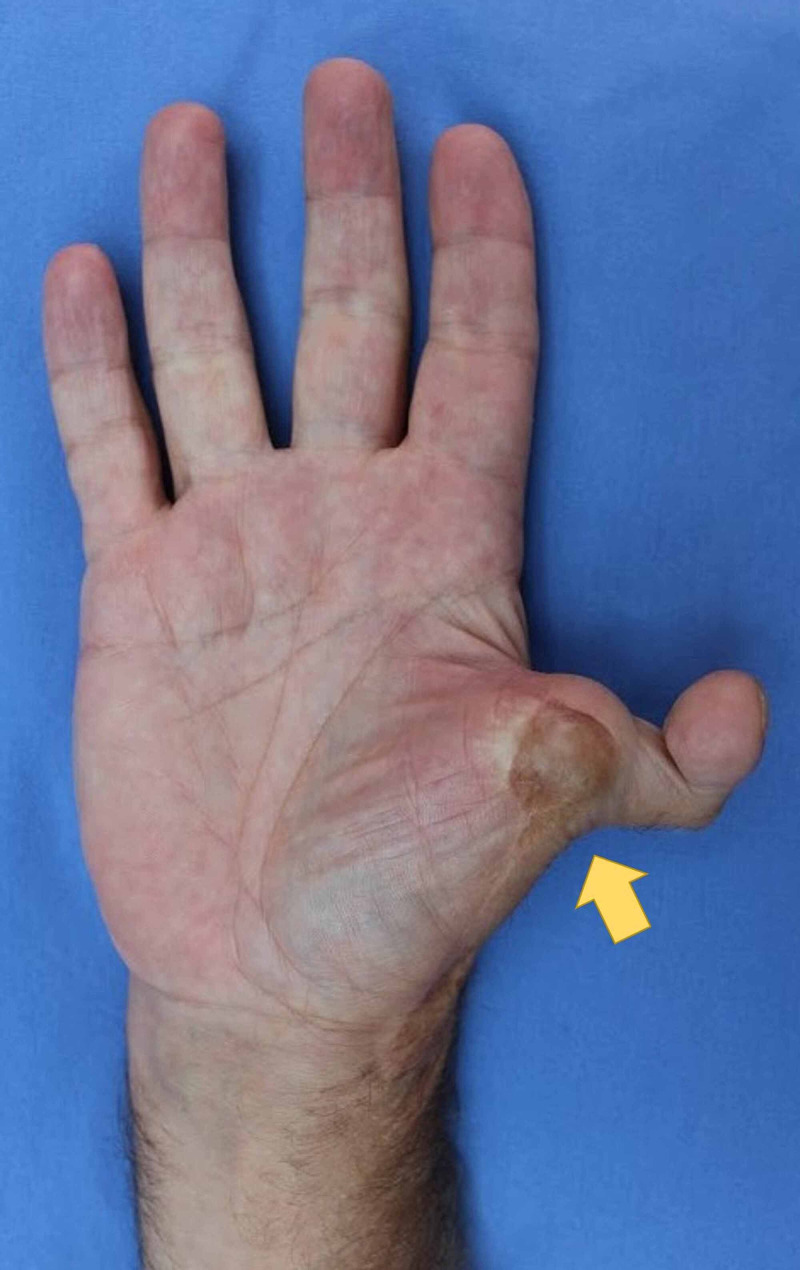
The ventral surface of the right hand, demonstrating the ventral aspect of the neothumb

**Figure 4:**
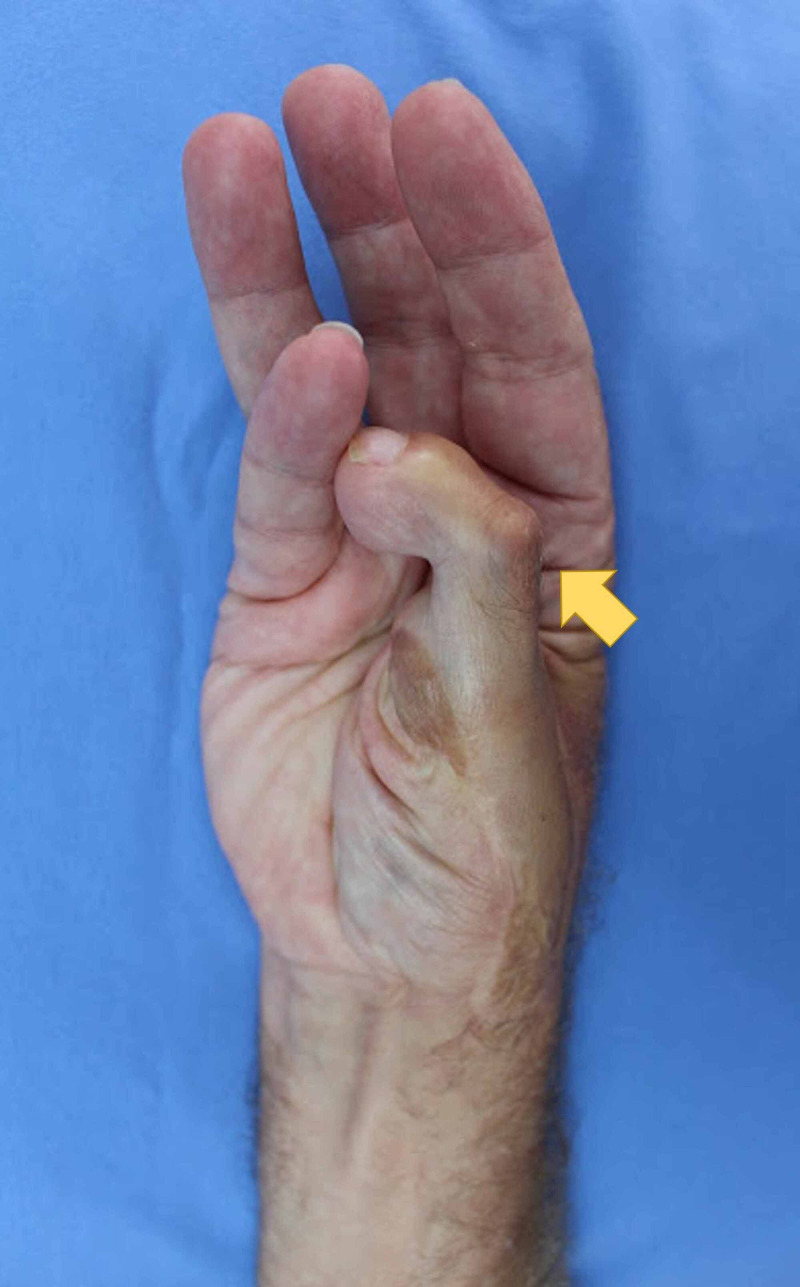
The Kapandji test being performed by the patient's right hand, scoring 7 out of 10

**Figure 5:**
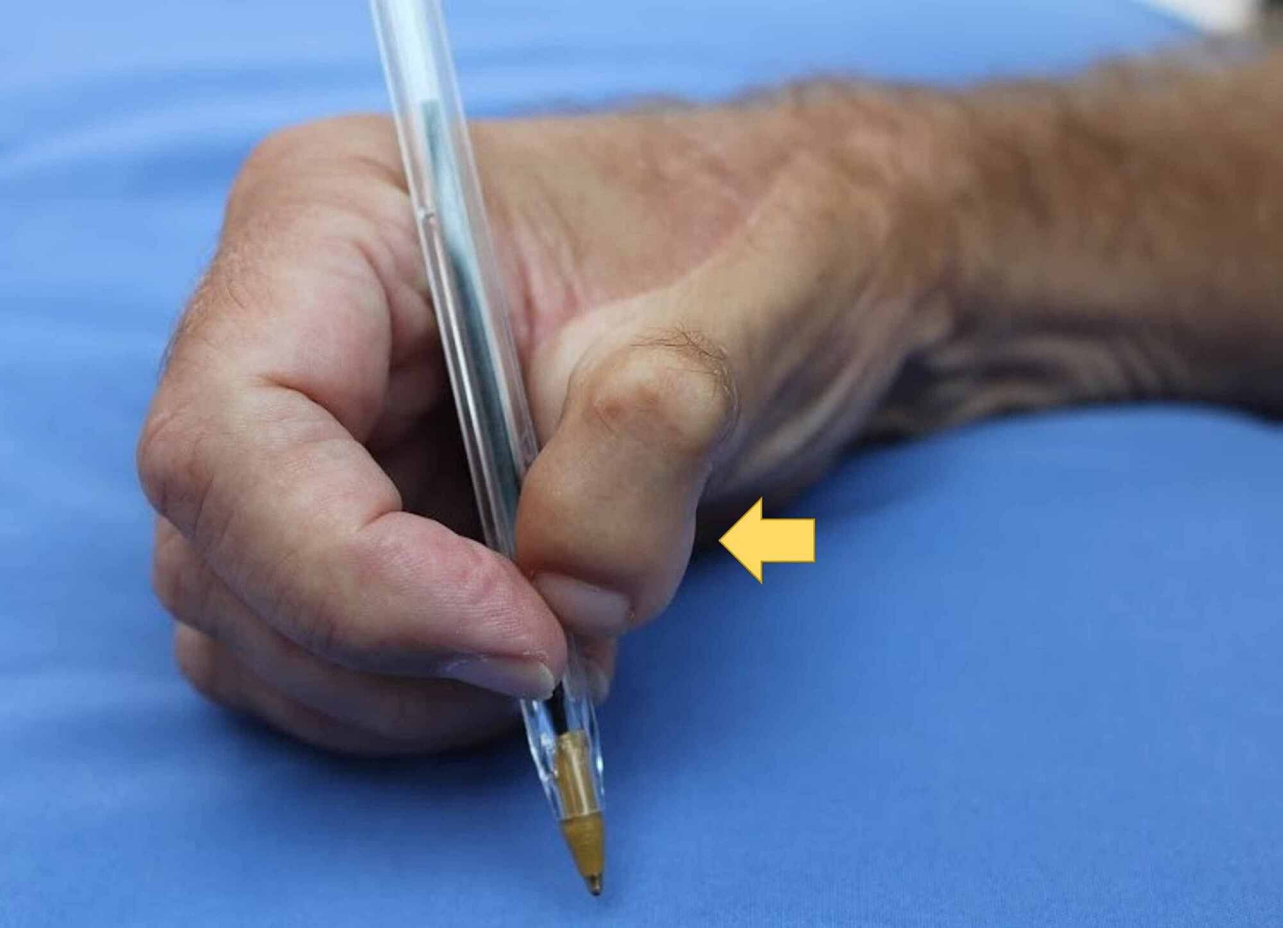
The patient's right hand holding a pen

**Figure 6:**
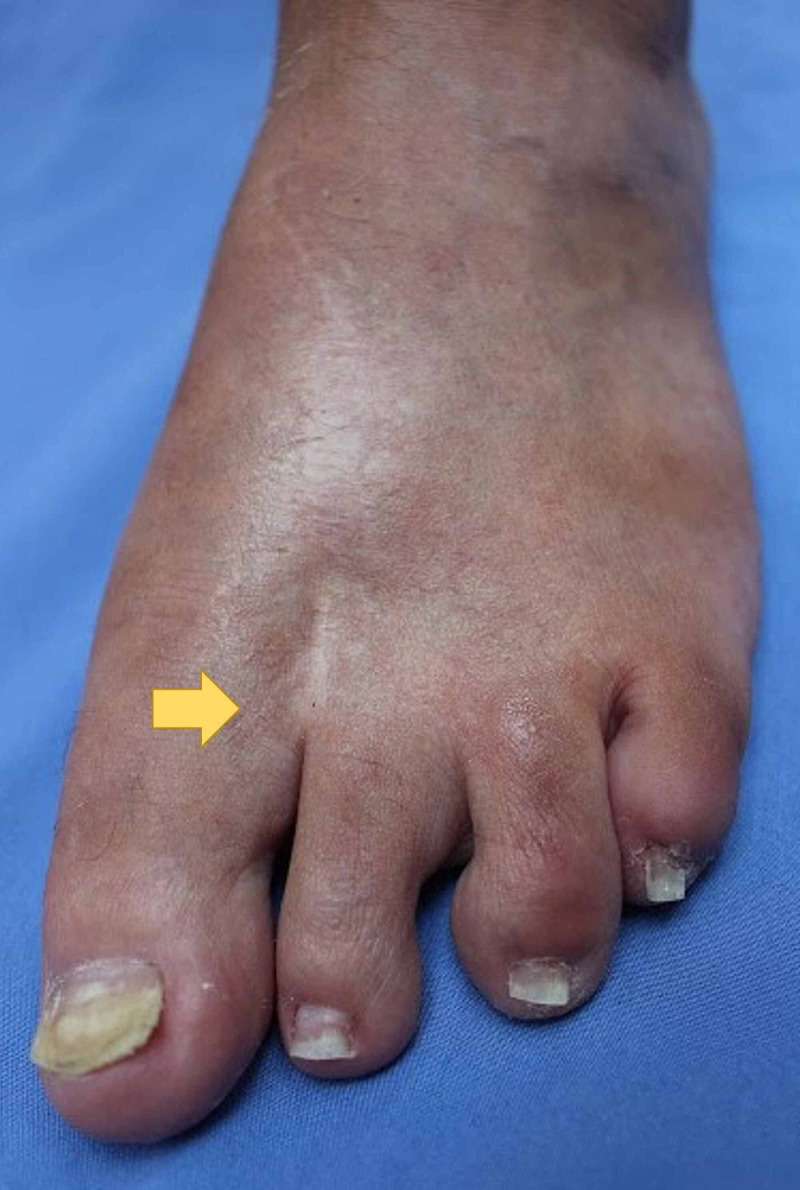
The patient's right foot, showing the index toe removed

Photography was used to document aesthetic outcomes. Figure 2 shows the dorsal surface of the right hand, demonstrating the dorsal aspect of the neothumb. Figure 3 shows the ventral surface of the right hand, demonstrating the ventral aspect of the neothumb. Figure 4 shows the patient performing the Kapandji test with their right hand, scoring seven out of 10. Figure 5 shows the dexterity of the patient's right hand, demonstrated by their ability to hold a pen. Figure 6 shows the patient's right foot from which the index toe was removed.

## Discussion

Thumb injuries are catastrophic for a patient’s function and hand aesthetics. Reconstructive surgery, alongside good hand rehabilitation, is shown to be effective in returning function to the hand. The preferred technique used to reconstruct the hand depends on the location and type of injury [8], the effect on the patient’s activities of daily living, and any further damage to the hand or other digits. The transfer of toes to hands has become well established, delivering good outcomes over many years [9]. However, there is no consensus over the correct approach with surgeons generally choosing to proceed with whatever technique they have been trained in. It is also worth noting that it is thought by some that index finger pollicisation gives greater sensation, improved appearance, and function. However, this is contraindicated if the palmar artery is injured or the other fingers are lost [10]. Other considerations when choosing the surgical approach is the immediacy of which the patient requires function to return. It is thought, although not proven conclusively, that index finger pollicization gives more immediate return of function over toe-thumb transfer. Hence, this approach should be considered in manual workers who may be on a limited income or are self-employed. It has also been discussed that it is not always necessary to restore full length of the thumb for good outcomes and that amputation of the thumb distal to the metacarpophalangeal (MCP) joint does not need to be reconstructed [11].

The basic elements of an adequate thumb reconstruction are to have a suitable length, a large web space, the sensation of the fingertip, and appropriate position. These combined with being able to oppose the fingers to pinch and grasp are the main determinants of positive outcomes [8, 12].

In this case, we present a second toe to thumb transfer that had very good outcomes after 34 years with few complications. As with many patients, our case demonstrates that great adaptation can occur over long periods of time accomplishing functions previously not thought possible. The patient was pleased with the aesthetic outcome of his neothumb, although he still experiences astonishment from new acquaintances on occasion. Functional outcomes as demonstrated by the patient’s scores were good with the main deficiencies in fine motor skills. The patient continues to have excellent function and stability in the donor foot, with some minor ongoing pain at the donor site.

## Conclusions

This case demonstrates that there can be good long term outcomes from toe to thumb transfer despite moderate impairment of function. It is hoped that this case can be used to encourage and reassure patients and surgeons alike that the neothumb is likely to give good outcomes, both functionally and aesthetically, for many years. 
